# Prophylactic-dose direct oral anticoagulants for non-hospitalised people with COVID-19: A meta-analysis of randomised controlled trials

**DOI:** 10.7189/jogh.14.05015

**Published:** 2024-04-26

**Authors:** Qingchun Song, Hongduan Liu, Haoyu Tan, Benli Yang, Hao Zhang, Liming Liu, Chengming Fan

**Affiliations:** Department of Cardiovascular Surgery, the Second Xiangya Hospital, Central South University, Changsha, Hunan, China

## Abstract

**Background:**

Several reviews have been conducted on thromboprophylaxis in non-hospitalised patients with coronavirus disease 2019 (COVID-19). In this systematic review and meta-analysis, we sought to investigate the impact of prophylactic-dose direct oral anticoagulants (DOACs) in this population.

**Methods:**

We searched PubMed, Web of Science, EMBASE and Cochrane Library for randomised controlled trials (RCTs) comparing prophylactic-dose DOACs with placebo or no treatment in non-hospitalised patients with COVID-19 until September 2023. The primary efficacy outcome was a composite of all-cause mortality and thromboembolic events, while major bleeding events were the primary safety outcome. We expressed continuous outcome data as mean differences (MDs) with 95% confidence intervals (CIs) and dichotomous outcome data as risk ratios (RRs) with 95% CIs.

**Results:**

We included six RCTs involving 4307 patients. Prophylactic-dose DOAC therapy compared with placebo or no treatment was associated with significantly decreased risks of the composite outcome of all-cause mortality and thromboembolic events (1.43% vs 2.67% (RR = 0.53; 95% CI = 0.34–0.82, *P* = 0.004, *I*^2^ = 3%)). Major bleeding events were infrequent, and we detected no significant differences between patients assigned to prophylactic-dose DOACs vs placebo or no treatment (0.19% vs 0.05% (RR = 2.50; 95% CI = 0.49-12.87, *P* = 0.27, *I*^2^ = 0%)). The use of prophylactic-dose DOACs was also associated with a reduction in venous thromboembolism, with no difference in all-cause mortality, arterial thromboembolism, hospitalisations, and clinically relevant nonmajor bleeding between two groups. Sensitivity analyses with the leave-one-out method for the primary efficacy and safety outcome did not change the effect estimate substantially.

**Conclusions:**

We found that prophylaxis-dose DOACs could significantly improve clinical outcomes and reduce venous thrombotic events without increasing the risk of major bleeding events compared with placebo or no treatment in non-hospitalised patients with COVID-19.

**Registration:**

PROSPERO: CRD42023466889.

The coronavirus disease 2019 (COVID-19) was previously found to be associated with a significantly higher risk of thromboembolic complications [1-3]. In COVID-19 patients, including those receiving outpatient care, ensuing macrovascular and microvascular thrombosis may contribute to clinical deterioration and fatalities [4-6]. Meanwhile, research has recommended prophylactic use of anticoagulants for patients hospitalised with COVID-19 [7,8]. Despite adequate thromboprophylaxis during hospitalisation, the risk of out-hospital thromboembolism remains high, with studies showing that the incidence of venous thromboembolism (VTE) is up to 2.5% [9]. Additionally, a substantial proportion of mildly infected COVID-19 patients do not require hospitalisation [4]. Direct oral anticoagulants (DOACs) have been used as pharmacological interventions to prevent thrombosis events, and their application in outpatient settings could decrease the incidence of vascular thrombosis and related mortality in individuals with COVID-19. However, clinicians need to consider potential adverse events, including bleeding, before initiating this treatment [10]. This leaves a need for a robust evaluation of the evidence in the use of anticoagulants in non-hospitalised patients with COVID-19 is imperative.

Current guidelines do not recommend the initiation of direct oral anticoagulant treatment for symptomatic COVID-19 patients not requiring hospitalization or the extension of thromboprophylaxis for post-discharge patients [11,12]. Nevertheless, these guidelines primarily rely on expert consensus rather than robust evidence. As of yet, several randomised clinical trials (RCTs) and observational studies have explored the effectiveness and safety of DOACs in non-hospitalised patients with COVID-19, with inconsistent findings [13-21]. A recent meta-analysis of RCTs compared DOACs and placebo in non-hospitalised patients with COVID-19 [22]. However, the small number of included studies and the variations in clinical settings (hospitalised participants included in one trial [23]) introduced potential bias. To date, there has been no meta-analysis that has specifically concentrated on evaluating the effects of prophylactic-dose DOACs in non-hospitalised COVID-19 patients. We sought to address this gap through a systematic review and meta-analysis.

## METHODS

We registered the protocol for this review in PROSPERO (CRD42023466889) and reported our findings according to the PRISMA guidelines.

### Data sources and search strategy

We searched PubMed, Embase, the Cochrane Library, and the Web of Science for articles published by 28 September 2023, without additional restrictions on the language or the year of publication. We designed our search strategy (Table S1 in the **Online Supplementary Document**) per the PICOS framework, with the two essential components being COVID-19 patients and DOACs.

### Study selection

We looked for RCTs assessing the efficacy and safety outcomes of prophylactic-dose DOACs compared to placebo or no treatment in non-hospitalised patients with COVID-19. Therefore, we included prospective RCTs with adult participants (aged ≥18 years), including outpatients and patients who were hospitalised with COVID-19 at discharge, and which had provided data on all-cause mortality, VTE, arterial thromboembolism (ATE), and/or major bleeding. We excluded reviews, case series, or observational studies; studies without a comparison group; and studies on inpatients with COVID-19.

Two researchers (QS and HL) reviewed titles and abstracts in NoteExpress, v3.9.0 (AegeanSoftware Corp, Beijing, China) using pre-defined criteria, followed by their full texts. They resolved disagreements by mutual consensus with a third researcher (HT).

### Data extraction and quality appraisal

Two researchers (QS and BY) extracted data from each study into RevMan, version 5.3 (Cochrane Collaboration, Copenhagen, Denmark) based on a structured data extraction form. This included the study characteristics (authors, publication year, study design, COVID-19 patient population, sample size, drugs for thromboprophylaxis, thromboprophylaxis duration, follow-up duration, primary outcome), the participants’ baseline characteristics (mean age, sex, hypertension, diabetes, body mass index, history of smoking, platelet count, D-dimer, COVID-19 vaccination, and the duration of index hospitalisation was extracted in post-discharge patients), intervention (drugs for thromboprophylaxis, drug dosage, duration of thromboprophylaxis), and outcome measures (all-cause mortality, VTE, ATE, hospitalisations, major bleeding, clinically relevant nonmajor bleeding).

Two researchers (QS and HL) evaluated the quality of each included trial using the first version of the Cochrane Risk of Bias tool [24]. Discrepancies in either stage were resolved by discussion with a third researcher (HT).

### Clinical outcomes

The primary efficacy outcome was a composite of all-cause mortality and thromboembolic events, while major bleeding was reported as the primary safety outcome. Secondary outcomes included all-cause mortality, VTE, ATE, hospitalisations, and clinically relevant nonmajor bleeding in both groups.

Thromboembolic events included VTE events (symptomatic or asymptomatic; first episode or recurrent deep venous thrombosis; pulmonary embolism; and thrombosis of other veins such as cerebral sinus and splanchnic veins) and ATE events (ischaemic stroke; myocardial infarction; and other arterial thrombosis such as mesenteric or acute limb ischaemia).

We defined major bleeding per the criteria of the International Society on Thrombosis and Haemostasis (ISTH) [12]. This includes fatal bleeding; symptomatic bleeding in critical areas or organs; intramuscular bleeding associated with compartment syndrome; a decrease in haemoglobin concentration of 2 g/dL or more; and transfusion of two or more units of whole blood or red cells.

### Statistical analysis

We performed a pairwise meta-analysis to compare prophylactic-dose DOACs against placebo in non-hospitalised patients with COVID-19. We presented dichotomous outcome data as risk ratios (RRs) with 95% confidence intervals (CIs) and continuous outcome data as mean differences (MDs) with 95% CIs. We assessed the log RRs for normality using the Shapiro-Wilk normality test when the number of effect estimates exceeded three. We evaluated the heterogeneity of the effect size across the studies using Cochran’s Q (*P* < 0.05 was considered heterogeneous) and *I*^2^ statistic (*I*^2^>25% indicated low heterogeneity, *I*^2^ = 25–50% indicated moderate heterogeneity, *I*^2^>0% indicated high heterogeneity). If no significant heterogeneity between studies was indicated, we used a fixed-effects model; otherwise, we used a random-effects model. To further investigate possible explanations for heterogeneity, we conducted a leave-one-out sensitivity analysis (i.e. excluded one study at a time and repeated the analysis) and a subgroup analysis by restricting studies to those enrolling outpatients or post-discharge patients with COVID-19, considering that the severity of the illness condition and the thromboprophylaxis regimen used during hospitalisation may influence the outcomes. We considered a *P*-value <0.05 as statistically significant.

We conducted all statistical analyses in SPSS, version 21.0 (IBM, Armonk, New York, USA) and RevMan, version 5.3 (Cochrane Collaboration, Copenhagen, Denmark).

## RESULTS

**Figure 1:**
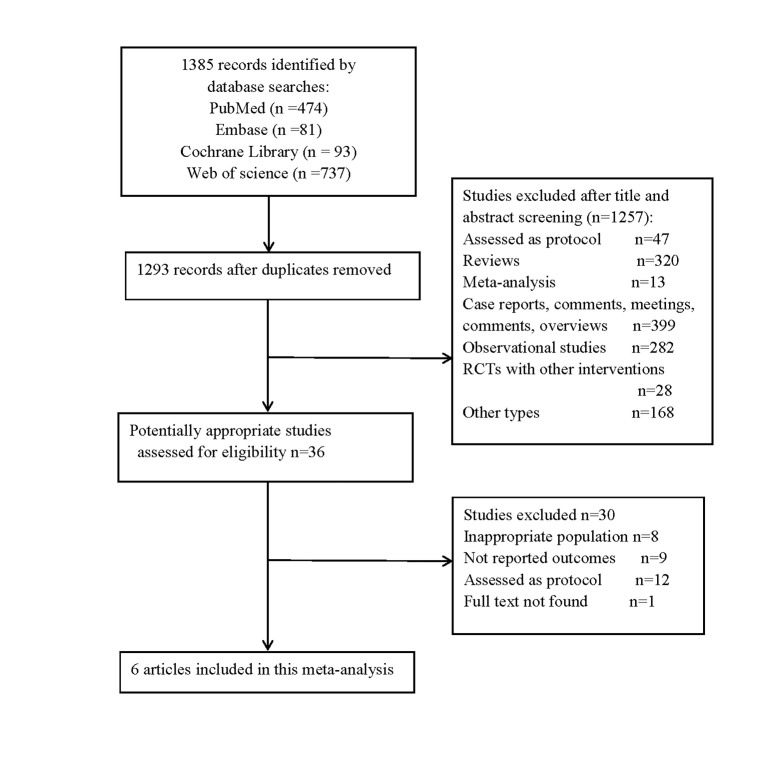
Selection process of included studies. RCT – randomised controlled trial.

The database search retrieved 1385 articles, with 1293 remaining after deduplication. After screening the titles, abstracts, and full texts, we included six RCTs in this analysis (**Figure 1**).

### Characteristics of the eligible trials

All studies were prospective, multicentre RCTs designed to assess the efficacy and safety of prophylactic-dose DOAC therapy in non-hospitalised patients with COVID-19. Four were conducted in the USA and two in Brazil. The COVID Antithrombotic Rivaroxaban Evaluation-Coalition VIII (CARE-COALITION VIII) [19] and Medically Ill hospitalized Patients for Covid - Thrombosis Extended Prophylaxis with rivaroxaban Therapy (MICHELLE) [18] trials were open-label, while the remaining four RCTs were double-blind [16,17,20,21]. All six trials used intention-to-treat analyses (**Table 1**).

**Table 1:** Characteristics of the included studies

Study, year	Study design	Country	COVID19 patient population	Drugs for thromboprophylaxis	Duration of thromboprophylaxis in days	Follow-up duration in days	Drug dosage	Primary outcome
ACTIV-4B, 2021 [16]	RCT	USA	Outpatient	Apixaban	45	75	2.5 mg, twice daily	Symptomatic DVT, PE, ATE, MI, ischaemic stroke, hospitalisation for cardiovascular or pulmonary events, and all-cause mortality.
Ananworanich et al, 2021 [17]	RCT	USA	Outpatient	Rivaroxaban	21	35	10 mg, once daily	The frequency of AEs and the proportion of participants who progressed to a moderate or severe disease.
MICHELLE, 2022 [18]	RCT	Brazil	Post-discharge	Rivaroxaban	35	35	10 mg, once daily	Symptomatic DVT, ATE, and major bleeding.
CARE – COALITION VIII, 2023 [19]	RCT	Brazil	Outpatient	Rivaroxaban	14	30	10 mg, once daily	VTE, need of mechanical ventilation, MACE, death not attributed to major injury within 30 d from randomisation, and hospitalisation.
PREVENT-HD, 2023 [20]	RCT	USA	Outpatient	Rivaroxaban	35	49	10 mg, once daily	Symptomatic VTE, MI, ischaemic stroke, acute limb ischemia, non-CNS systemic ATE, all-cause hospitalisation, all-cause mortality, and major bleeding.
ACTIV-4C, 2023 [21]	RCT	USA	Post-discharge	Apixaban	3	90	2.5 mg, twice daily	All-cause mortality, venous thrombosis, and arterial thrombosis, and major bleeding.

AE – adverse event, ATE – arterial thromboembolism, CNS – central nervous system, DVT – deep venous thrombosis, MACE – major adverse cardiovascular events, MI – myocardial infarction, PE – pulmonary embolism, RCT – randomised controlled trial, VTE – venous thromboembolism

The trials included 4307 participants, with 2150 randomised to DOAC therapy and 2157 to placebo or no treatment (**Table 2**). Their mean age ranged from 49 to 61 years. Among the participants, 2770 (64.3%) were outpatients and 1537 (35.7%) were post-discharge patients. The median body mass index ranged from 29.6 to 33.6 kg/m2. Moreover, 26.8% had diabetes and 53.2% reported a history of hypertension. These trials included patients with or without a high risk of VTE. COVID-19 vaccination was only reported in PREVENT-HD [20], where the vaccination rate was 2.1%. The patient selection criteria for each study are provided in Table S2 in the **Online Supplementary Document**.

**Table 2:** Baseline characteristics of clinical trials

Variable (treatment vs placebo)	ACTIV-4B, 2021 [16]	Ananworanich et al, 2021 [17]		MICHELLE, 2022 [18]	CARE – COALITION VIII, 2023 [19]	PREVENT-HD, 2023 [20]	ACTIV-4C, 2023 [21]
Age in years, x̄ (SD)	55.0 (46.0–61.0) vs 54.0 (45.0–59.0)*		49 (20.0–83.0) vs 49 (18.0–75.0)†	57.8 (14.8) vs 56.4 (15.6)‡	61.0 (49.0–69.0) vs 60.0 (46.0–69.0)*	56.3 (13.1) vs 55.7 (13.3)‡	54.0 (44.0–64.0) vs 54.0 (44.0–64.0)*
Male, n (%)	70 (42.4) vs 68 (41.5)		96 (43.2) vs 81 (36.5)	97 (61%) vs 94 (59%)	140 (42.5) vs 152 (45.9)	242 (37.8) vs 259 (40.3)	299 (49.9) vs 304 (50.1)
Number of randomized individuals	164 vs 165		246 vs 251	160 vs 160	329 vs 331	641 vs 643	610 vs 607
Total follow-up in days	45		35	35	30	49	90
Body mass index, MD (IQR)	29.9 (26.2–34.8) vs 30.3 (26.5–34.7)*		35.2(16.9–68.7) vs 33.2(18.8–66.4)†	29.6 (5.6) vs 29 · 9 (6.0)‡	31.2 (27.3–34.3) vs 30.8 (27.5–34.9)*	33.6 (7.9) vs 33.6 (8.15)‡	32.7 (27–39) vs 32.9 (28–39)*
Hypertension, n (%)	66 (40.0) vs 54 (32.9)		106 (47.7) vs 124 (55.9)	NA	259 (78.7) vs 262 (79.2)	NA	294 (48.2) vs 275 (45.3)
Diabetes, n (%)	36 (21.8) vs 24 (14.6)		57 (25.7) vs 66 (29.7)	54 (50.5) vs 56 (50.5)	113 (34.3) vs 122 (36.9)	141 (22) vs 133 (20.7)	180 (29.5) vs 165 (27.2)
History of smoking, n (%)	29 (17.6) vs 31 (18.9)		NA	NA	NA	229 (35.8) vs 203 (31.6)	93 (15.2) vs 86 (14.2)
Platelet count in mm3	250.0 (187–295) vs 238.0 (189–319)*		NA	NA	NA	NA	306.0 (239–395) vs 313.0 (225–405)*
D-dimer >1ULN, n (%)	53 (34.9) vs 52 (33.0)		NA	106 (92.0) vs 108 (92.0)	NA	NA	334 (54.8) vs 359 (59.1)
Duration of index hospitalisation in days	NA		NA	8 (5.5-12) vs 8 (6-12)*	NA	NA	6 (5–9) vs 6 (5–9)*
COVID-19 vaccination, n (%)	NA		NA	NA	NA	16 (2.5) vs 11 (1.7)	NA

NA – not applicable, ULN – upper limit of normal

*Values expressed as medians with interquartile ranges.

†Values expressed as medians with minimum and maximum values.

‡Values expressed as means with standard deviations.

Regarding risk of bias (Figure S1 in the **Online Supplementary Document**), Ananworanich et al. [17] and the Accelerating COVID-19 Therapeutic Interventions and Vaccines-4C (ACTIV-4C) [21] trial did not report on their random sequence generation method, so we judged them to have unclear risk of bias. Blinding of participants and study personnels was not feasible in the CARE-COALITION VIII [19] and MICHELLE [18] trials. We judged the remaining trials to be at low risk of bias in all the other domains.

DOACs given in the included trials were rivaroxaban or apixaban. Prophylactic-dose apixaban (2.5 mg twice daily) was received in Accelerating COVID-19 Therapeutic Interventions and Vaccines-4B (ACTIV-4B) [16] for 45 days and ACTIV-4C [21] for 30 days. The other four trials administered prophylactic-dose rivaroxaban (10 mg daily) with varying durations: 35 days in MICHELLE [18] and PREVENT-HD [20], 14 days in CARE-COALITION VIII [19], and 21 days in Ananworanich et al. [17].

The results of the Shapiro-Wilk normality test showed that the outcomes extracted from the included trails followed a normal distribution (Table S3 in the **Online Supplementary Document**).

### Primary outcome

**Figure 2:**
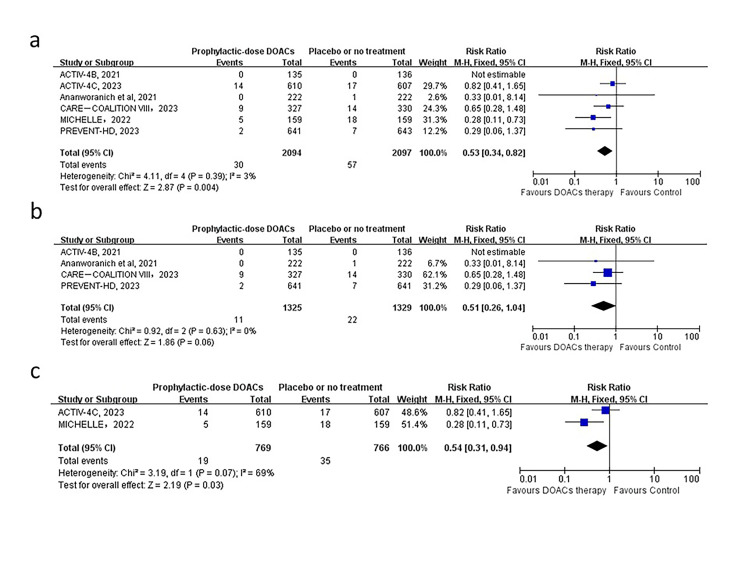
Forest plot of the association of prophylactic-dose DOACs with composite outcome of all-cause mortality and thromboembolic events in non-hospitalised patients with COVID-19. **Panel A**. Analysis in non-hospitalised patients. **Panel B**. Subgroup analysis in outpatients. **Panel C.** Subgroup analysis in post-discharge patients. ATE – arterial thromboembolism, DOAC – direct oral anticoagulant, VTE – venous thromboembolism.

**Figure 3:**
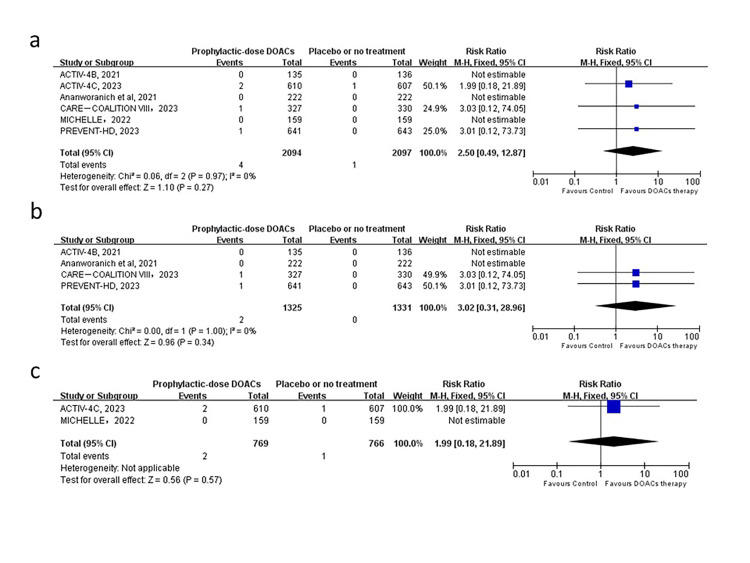
Forest plot of the association of prophylactic-dose DOACs with major bleeding in non-hospitalised patients with COVID-19. **Panel A**. Analysis in non-hospitalised patients. **Panel B**. Subgroup analysis in outpatients. **Panel C.** Subgroup analysis in post-discharge patients. DOAC – direct oral anticoagulant.

In the six included trials, we found that prophylactic-dose DOAC therapy, when compared with placebo, significantly reduced the risk of the primary efficacy outcome (1.43% vs 2.67% (RR = 0.53; 95% CI = 0.34–0.82, *P* = 0.004, *I*^2^ = 3%)) (**Figure 2**, Panel A). There was no substantial heterogeneity, and the leave-one-out sensitivity analysis showed no significant alteration in the effect estimate (Table S4 in the **Online Supplementary Document**). The subgroup analyses showed significant differences in post-discharge patients, but none in outpatients (**Figure 2**, Panels B and C). The primary safety outcome was major bleeding, which was documented in all the trials in our analysis. Major bleeding events were infrequent and occurred in four patients receiving prophylactic-dose DOACs and one receiving placebo or no treatment. There was no significant difference between patients assigned to DOACs vs placebo and no statistical evidence of heterogeneity among the trials (0.19% vs 0.05%; (RR = 2.50; 95% CI = 0.49–12.87, *P* = 0.27, *I*^2^ = 0%)) (**Figure 3**, Panel A). The leave-one-out sensitivity analysis showed no substantial alteration in the effect estimate (Table S5 in the **Online Supplementary Document**). Subgroup analysis of studies with major bleeding in outpatients or post-discharge patients did not reveal any substantial change (**Figure 3**, Panels B and C).

### Secondary outcomes

All the trials in our analysis reported all-cause mortality, with rates similar in both the DOAC and the no treatment groups without substantial heterogeneity (0.76% vs 1.14% (RR = 0.67; 95% CI = 0.36–1.25, *P* = 0.21, *I*^2^ = 0%)). Thromboembolic events (including VTE and ATE events) were reported in all trials except for Ananworanich et al. [17]. Prophylactic-dose DOACs were associated with significantly decreased risks of the VTE events compared with placebo or no treatment without substantial heterogeneity (0.53% vs 1.33% (RR = 0.42; 95% CI = 0.21–0.85, *P* = 0.02, *I*^2^ = 6%)). The risk of ATE events was not significantly different between patients randomised to DOAC therapy vs placebo and there was no statistical evidence of heterogeneity among the trials (0.21% vs 0.37% (RR = 0.63; 95% CI = 0.21–1.91, *P* = 0.41, *I*^2^ = 0%)) (Figure S2–4 in the **Online Supplementary Document**).

Hospitalisations were reported in four trials [16,17,19,20] which exclusively included outpatients. There was no significant difference between DOACs and placebo in the rate of hospitalisations, without substantial heterogeneity (4.6% vs 4.2% (RR = 1.10; 95% CI = 0.77–1.55, *P* = 0.61, *I*^2^ = 0%)) (Figure S5 in the **Online Supplementary Document**).

Five trials reported events of clinically relevant nonmajor bleeding. We observed that prophylactic-dose DOACs were not associated with a significant increase in clinically relevant nonmajor bleeding events, and there was no statistical evidence of heterogeneity among the trials (1.13% vs 0.62% (RR = 1.78; 95% CI = 0.87–3.66, *P* = 0.12, *I*^2^ = 36%)) (Figure S6 in the **Online Supplementary Document**).

## DISCUSSION

To our knowledge, this is the first meta-analysis of RCTs on the effectiveness and safety of prophylactic-dose DOACs in non-hospitalised patients with COVID-19. We found that treatment with prophylactic-dose DOACs significantly reduced the incidence of the composite efficacy outcome of all-cause mortality and thromboembolic events in non-hospitalised patients with COVID-19 without increasing the risk of major bleeding events, and that the treatment group had lower risk of VTE events than the non-treatment group, without a significant difference in all-cause mortality, ATE events, hospitalisations, and clinically relevant nonmajor bleeding. This indicates that DOACs can improve clinical outcomes without increasing the risk of bleeding events in non-hospitalised patients with COVID-19.

Although previous research has shown an increase in cases of thromboembolic events among patients with COVID-19 not requiring hospitalisation [25], there is currently no definitive consensus regarding the impact of prophylactic anticoagulants in managing outpatients with COVID-19, and the effects of prophylactic anticoagulants are still being discussed. The identification of effective anticoagulant interventions is crucial for lowering COVID-19 hospitalisation rates and associated complications, including thromboembolic events and mortality. Thus, there is a pressing need for evidence to inform treatment strategies for non-hospitalised patients with COVID-19. To our knowledge, several reviews have focused on thromboprophylaxis in non-hospitalised patients with COVID-19 [22,26,27]. A recent meta-analysis suggested that prophylactic anticoagulants may decrease the incidence of VTE and PE when compared to placebo or no treatment in non-hospitalised individuals. However, this approach may not fully address our concerns, since DOACs were used in only two of the trials [26]. Another meta-analysis of 1874 patients from four RCTs indicated that thromboprophylaxis with DOACs in non-hospitalised patients improved clinical outcomes and reduced thrombotic events when compared to no anticoagulation [22]. However, the result was limited by the small sample size in the included trials and an indirect comparison between DOACs vs placebo. Moreover, this review included one trial with hospitalised patients [23], which we excluded from this review to decrease the bias in the analysis.

The six trials included in our analysis compared DOACs with placebo or no treatment and investigated our four outcomes of interest (the composite efficacy outcome, all-cause mortality and major bleeding) during short-term follow-up (30-90 days). Ananworanich et al did not document thromboembolic events. Moreover, the data extracted from the MICHELLE [18] and CARE-COALITION VIII [19] trials should be interpreted with caution, given the high risk of bias for the blinding of participants and personnel domain. Our analysis showed that prophylactic-dose DOAC therapy is more effective than placebo or no treatment in non-hospitalised patients with COVID-19. Notably, of all the included trials, only MICHELLE concluded that thromboprophylaxis with rivaroxaban 10 mg daily through 35 days improved clinical outcomes and reduced thrombotic events compared with no treatment in post-discharge patients. Other trials demonstrated little or no impact of rivaroxaban on clinical outcomes in patients with COVID-19 or were inconclusive. These inconsistencies might be explained by several factors. First, three trials (ACTIV-4B, PREVENT-HD, and ACTIV-4C) were terminated early given lower-than-expected primary event rate and slow enrolment, which made the result imprecise and the study underpower, meaning it might have missed significant differences in efficacy or safety [16,20,21]. Second, all included trials were characterised by lower-than-planned sample sizes and lower-than-expected event rates. For example, no primary efficacy outcomes were found in ACTIV-4B [16] and only one was detected in Ananworanich et al. [17]. Although events were marginally less frequent in the DOACs group of the PREVENT-HD [20] and CARE-COALITION VIII [19] trials, the nominal number of events and statistical analysis precluded the interpretation of a treatment benefit of DOACs. Lastly, despite the prophylactic dose of DOACs being consistent in all the trials (rivaroxaban 10mg daily or apixaban 2.5 mg twice daily), the duration of treatment varied significantly from 14 to 45 days, which may have further contributed to the inconsistencies in the results.

Safety outcomes are another significant consideration in the thromboprophylaxis treatment of COVID-19 patients. In this meta-analysis, we included major bleeding and clinically relevant nonmajor bleeding events as our safety outcome. Prior studies have demonstrated that prophylactic doses are associated with a lower risk of major bleeding events compared to therapeutic doses [28]. In our study, major bleeding events were infrequent. Only four patients received prophylactic-dose NOACs, and one received placebo in three trials, with no cases detected in the other trials. Consequently, we found that prophylactic-dose DOACs did not result in a significant difference in major bleeding compared to placebo or no treatment. However, considering the wide confidence interval (95% CI = 0.49–12.87), larger samples are needed to further validate this result. Moreover, with an RR of 2.50, major bleeding events should not be overlooked when assessing the benefits and risks of thromboprophylaxis in non-hospitalised patients with COVID-19. Five RCTs reported on clinically relevant nonmajor bleeding events (i.e. except for the CARE-COALITION VIII trial [19]), without a significant difference between the two groups. Nevertheless, prior analyses have shown inconsistent results [22] which could be attributed to variations in the types and doses of anticoagulant interventions or differences in the settings of their population.

There was no significant heterogeneity in our primary outcomes, with the leave-one-out sensitivity analyses having no substantial influence on the effect estimate. However, several factors which were not detect in our analysis might contribute to heterogeneity and could impact our findings, including the setting of the intervention and follow-up time point; the ages of patients; the severity of illness; vaccination rate; the circulation of different severe acute respiratory syndrome (SARS-CoV-2) variants; comorbidities; or concomitant medications. Furthermore, our subgroup analysis suggested that prophylactic-dose DOAC therapy was not significantly associated with less frequent composite efficacy events compared with placebo or no treatment in the outpatient setting, which is inconsistent with the primary analysis. The small number of participants in each subgroup might have contributed to these detected differences; future trials with larger sample sizes are warranted to confirm these findings.

Our results should be interpreted with caution in the context of clinical practice due to several reasons. First, in our analysis, a large portion of participants had comorbidities, including obesity, hypertension, diabetes and some other diseases, which were previously found to be associated with a high risk of thromboembolism or clinical deterioration [29]. Moreover, concomitant medications, such as the use of an antiplatelet agent in these patients, may have also impacted the thrombotic and bleeding events. Thus, thromboprophylaxis may not be generalised to all patients (especially younger, healthier populations) with COVID-19. Second, the RCTs included this analysis were predominantly conducted in the USA and Brazil. Healthcare levels and patient demographics in different areas can interfere with the acceptability and effectiveness of treatment for patients. The outcomes should therefore be interpreted appropriately in different health care settings. Third, the circulation of different SARS-CoV-2 variants may be correlated with variations in the severity of illness and with varying risks of thrombotic events [30,31]. Since the RCTs in our analysis were conducted between 2021 and 2023, covering periods when the original, Alpha, Delta, and Omicron variants were circulating, the lack of information on the prevalence rates of different COVID-19 variants could affect the generalisability of the findings.

### Limitations

Our analysis has several limitations. First, the open-label design in the MICHELLE and CARE-COALITION VIII trials might have contributed to the observed benefits of the DOAC. Second, although we set restrictions on drug type and dosage, the duration of DOAC intervention and follow-up showed varied considerably. A previous meta-analysis suggested that extended-duration (28–35 days) thromboprophylaxis reduced the risk of deep venous thrombosis and pulmonary embolism, but was associated with an increased risk of major bleeding compared to short-term thromboprophylaxis (6–14 days) in acutely ill hospitalised medical patients. Therefore, the broad range of DOAC intervention durations (14–45 days) in the included trials may have impacted the efficacy and safety outcomes. Moreover, the short follow-up duration meant we had no data on possible later complications in patients [32]. Third, the introduction of vaccines for COVID-19 was associated with a milder disease course and a decreased risk of hospitalisation and death [33,34]. A substantial proportion of patients in ACTIV-4B and PREVENT-HD had not received vaccination before randomisation, while the information regarding vaccination rate was unavailable in other trials. Considering the currently evolving vaccination strategies, the generalisability of our findings to patients with COVID-19 vaccination may be limited. Fourthly, our analysis encompassed two distinct settings for COVID-19 patients: Post-discharge patients (i.e. after hospitalisation) and outpatients. Although we conducted subgroup analyses to address the potential heterogeneity, the limited data on each subgroup may not provide a comprehensive understanding of COVID-19 in post-discharge patients and outpatients. Finally, like all other meta-analyses, ours is susceptible to the effect of publication bias and other biases, as well as the limitations of the data reported in the included RCTs.

## CONCLUSIONS

Our meta-analysis of RCTs showed that prophylaxis-dose DOAC therapy could significantly improve clinical outcomes and reduce venous thrombotic events without increasing the risk of major bleeding events compared with placebo or no treatment in non-hospitalised patients with COVID-19.

## Additional material


Online Supplementary Document
