# Supplementary material for: Prophylactic-dose direct oral anticoagulants for non-hospitalised people with COVID-19: A meta-analysis of randomised controlled trials
Source: J Glob Health. 2024 Apr 26;14:05015. doi: 10.7189/jogh.14.05015 (PMC11046256; doi:10.7189/jogh.14.05015)
Supplement: Online Supplementary Document [file jogh-14-05015-s001.pdf]

ONLINE SUPPLEMENTARY DOCUMENT

Title: Prophylactic-dose direct oral anticoagulants for non-hospitalised people with COVID-19: A meta-analysis of randomised controlled trials

Authors: Qingchun Song, Hongduan Liu, Haoyu Tan, Benli Yang, Hao Zhang, Liming Liu, Chengming Fan

Table S1. The search details
Table S2. Patient selection for DOAC therapy
Table S3. The results of the Shapiro-Wilk test with effect estimates included in the analysis
Table S4. Sensitivity analysis with the leave-one-out method for the primary efficacy outcome
Table S5. Sensitivity analysis with the leave-one-out method for the primary safety outcome
Figure S1. Risk of bias assessment for included studies
Figure S2. Forest plot illustrating the association of prophylactic-dose DOACs with all-cause mortality in non-hospitalized patients with COVID-19
Figure S3. Forest plot illustrating the association of prophylactic-dose DOACs with VTE events in non-hospitalized patients with COVID-19
Figure S4. Forest plot illustrating the association of prophylactic-dose DOACs with ATE events in non-hospitalized patients with COVID-19
Figure S5. Forest plot illustrating the association of prophylactic-dose DOACs with hospitalizations in non-hospitalized patients with COVID-19
Figure S6. Forest plot illustrating the association of prophylactic-dose DOACs with clinically relevant nonmajor bleeding events in non-hospitalized patients with COVID-19

Table S1. The search details

Table S1a. Search strategy with PubMed

**PubMed** (Date of most recent search, September 28, 2023)		
**Number**	**Searches**	**Results**
#1	" direct oral anticoagulant "[MeSH Terms] OR " direct oral anticoagulant "[Title/Abstract] OR " DOAC "[Title/Abstract] OR " NOAC "[Title/Abstract] OR " non-vitamin K antagonist oral anticoagulant "[Title/Abstract] OR " novel oral anticoagulant "[Title/Abstract] OR " new oral anticoagulant "[Title/Abstract] OR " rivaroxaban "[Title/Abstract] OR " apixaban "[Title/Abstract] OR " edoxaban "[Title/Abstract] OR " dabigatran "[Title/Abstract] OR " betrixaban "[Title/Abstract] OR " Xa inhibitor "[Title/Abstract]	30716
#2	"COVID-19"[MeSH Terms] OR "COVID-19 "[Title/Abstract] OR "COVID19"[Title/Abstract] OR "2019 novel coronavirus infection "[Title/Abstract] OR "coronavirus disease 19"[Title/Abstract] OR "2019 novel coronavirus disease"[Title/Abstract] OR " Coronavirus*"[Title/Abstract] OR " Deltacoronavirus*"[Title/Abstract] OR " Delta-coronavirus*"[Title/Abstract]	371721
#3	#1 AND #2	474

Table S1b. Search strategy with Web of Science

**Web of Science** (Date of most recent search, September 28, 2023)		
**Number**	**Searches**	**Results**
#1	((((((((((((TS=(direct oral anticoagulant)) OR TS=(direct oral anticoagulants)) OR TS=(DOAC)) OR TS=(NOAC)) OR TS=(non-vitamin K antagonist oral anticoagulant)) OR TS=(novel oral anticoagulant)) OR TS=(new oral anticoagulant)) OR TS=(rivaroxaban)) OR TS=(apixaban)) OR TS=(edoxaban)) OR TS=(dabigatran)) OR TS=(betrixaban)) OR TS=(Xa inhibitor)	44489
#2	(((((((TS=(COVID-19)) OR TS=(COVID19)) OR TS=(2019 novel coronavirus infection)) OR TS=(coronavirus disease 19)) OR TS=(2019 novel coronavirus disease)) OR TS=(Coronavirus*)) OR TS=(Deltacoronavirus*)) OR TS=(Delta-coronavirus*)	621228
#3	#1 AND #2	737

Table S1c. Search strategy with Embase

**Embase** (Date of most recent search, September 28, 2023)		
**Number**	**Searches**	**Results**
#1	'direct oral anticoagulant':ti OR 'DOAC':ti OR 'NOAC':ti OR ' non-vitamin K antagonist oral anticoagulant ':ti OR ' novel oral anticoagulant ':ti ' new oral anticoagulant ':ti OR ' rivaroxaban ':ti OR ' apixaban ':ti OR ' edoxaban ':ti OR ' dabigatran ':ti OR ' betrixaban ':ti OR ' Xa inhibitor ':ti	13953
#2	' COVID-19':ti OR ' COVID19':ti OR '2019 novel coronavirus infection ':ti OR ' coronavirus disease 19 ':ti OR ' 2019 novel coronavirus disease ':ti OR ' Coronavirus* ':ti OR ' Delta-coronavirus* ':ti OR ' Deltacoronavirus* ':ti	284065
#3	#1 AND #2	81

Table S1d. Search strategy with Cochrane Library

Cochrane Library (Date of most recent search, September 28, 2023)

Number	Searches	Results
#1	(direct oral anticoagulant)ti,ab,kw OR (DOAC)ti,ab,kw OR (NOAC)ti,ab,kw OR (non-vitamin K antagonist oral anticoagulant)ti,ab,kw OR (novel oral anticoagulant)ti,ab,kw OR (new oral anticoagulant) ti,ab,kw OR (rivaroxaban)ti,ab,kw OR (apixaban)ti,ab,kw OR (edoxaban)ti,ab,kw OR (dabigatran)ti,ab,kw OR (betrixaban)ti,ab,kw OR (Xa inhibitor)ti,ab,kw	6089
#2	(COVID-19)ti,ab,kw OR (COVID19)ti,ab,kw OR (2019 novel coronavirus infection)ti,ab,kw OR (coronavirus disease 19)ti,ab,kw OR (2019 novel coronavirus disease)ti,ab,kw OR (Coronavirus*)ti,ab,kw OR (Delta-coronavirus*)ti,ab,kw OR (Deltacoronavirus*)ti,ab,kw	20129
#3	#1 AND #2	93

Table S2. Patient selection for DOAC therapy

Study	Patient selection
ACTIV-4B, 2021	Patients between the ages of 40 and 80 years with newly diagnosed symptomatic SARS-CoV-2 infection with positive polymerase chain reaction or antigen test results were eligible.
MICHELLE, 2022	Patients have an increased risk for venous thromboembolism, defined as an elevated modified IMPROVE VTE score of 2–3 with a D-dimer level of more than 500 ng/mL using local laboratory criteria or a score of 4 or more independent of the D-dimer level at hospital discharge.
Ananworanich et al, 2021	Patients with mild COVID-19 at screening and high risk for severe COVID-19 (either aged ≥65 years and diagnosed with a chronic disease that requires daily treatment such as diabetes, lung disease, heart disease, hypertension, or cancer or self-reported obesity).
CARE-COALITION VIII, 2023	Patients aged ≥18 years with suspected or confirmed COVID-19 of mild or moderate severity, presenting within ≤7 days from symptom onset. In addition, at least two of the following risk factors for clinical deterioration were required for eligibility: age >65 years, hypertension diabetes mellitus, asthma, COPD or other chronic lung diseases, current smoking, immunosuppression, obesity (defined as BMI ≥ 30 kg/m^2), history of non-active cancer, bedridden patient or with reduced mobility (cannot walk ≥50% of the awake time), previous history of VTE, or use of oral hormonal contraception.
PREVENT-HD, 2023	Patients were required to be at least 18 years of age and to have polymerase chain reaction or antigen-confirmed SARS-CoV-2 infection, symptomatic COVID-19, an initial treatment plan not including hospitalization, and at least one thrombosis risk factor:
ACTIV-4C, 2023	Potential study participants were older than 18 years and hospitalized for 48 hours or longer with a SARS-CoV-2 infection (positive polymerase chain reaction, antigen, or point-of-care test results within 2 weeks of the hospital admission date).

SARS-CoV-2, Severe acute respiratory syndrome coronavirus 2; VTE, Venous thromboembolism; COPD, chronic obstructive pulmonary disease; BMI, Body Mass Index

Table S3. The results of the Shapiro-Wilk test with effect estimates included in the analysis

**Outcomes**	**W**	***P* value**
Composite outcome	0.829	0.137
All-cause mortality	0.768	0.056
VTE events	0.924	0.558
ATE events	0.788	0.082
Hospitalizations	0.808	0.117
Clinically relevant nonmajor bleeding events	0.980	0.935

VTE, venous thromboembolism; ATE, arterial thromboembolism.

Table S4. Sensitivity analysis with the leave-one-out method for the primary efficacy outcome

**Study Omitted**	**RR (95% CI)**	**I^2**
ACTIV-4B, 2021	0.53 (0.34,0.82)	3%
Ananworanich et al, 2021	0.54 (0.35,0.83)	25%
MICHELLE, 2022	0.65 (0.40, 1.04)	0%
CARE-COALITION VIII, 2023	0.49 (0.30, 0.82)	23%
PREVENT-HD, 2023	0.57 (0.36, 0.89)	11%
ACTIV-4C, 2023	0.41 (0.23, 0.72)	0%

RR, Risk ratio; CI, confidence interval.

Table S5. Sensitivity analysis with the leave-one-out method for the primary safety outcome

**Study Omitted**	**RR (95% CI)**	**I^2**
ACTIV-4B, 2021	2.50 (0.49, 12.87)	0%
Ananworanich et al, 2021	2.50 (0.49, 12.87)	0%
MICHELLE, 2022	2.50 (0.49, 12.87)	0%
CARE-COALITION VIII, 2023	2.33 (0.34, 15.74)	0%
PREVENT-HD, 2023	2.33 (0.35, 15.76)	0%
ACTIV-4C, 2023	3.02 (0.31, 28.96)	0%

RR, Risk ratio; CI, confidence interval.

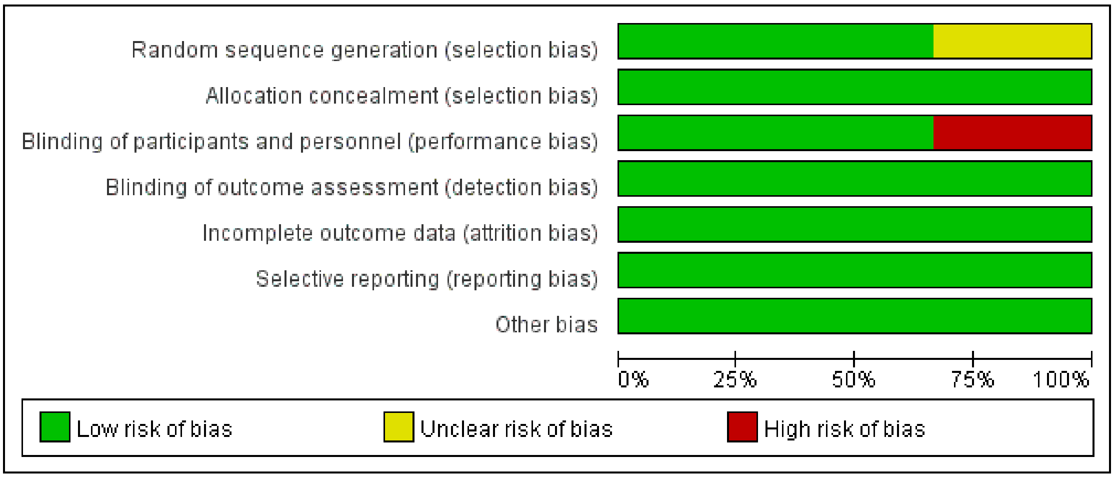

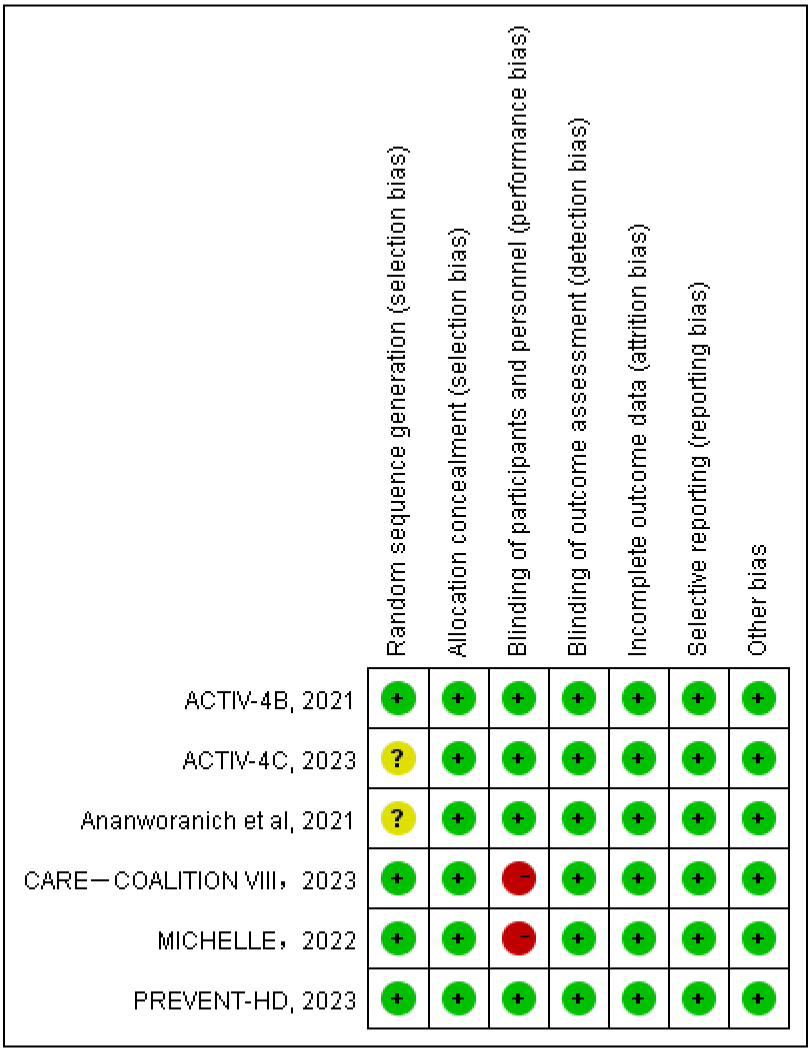

Figure S1. Risk of bias assessment for included studies

Study or Subgroup	Prophylactic-dose DOACs Events	Prophylactic-dose DOACs Total	Placebo or no treatment Events	Placebo or no treatment Total	Weight	Risk Ratio M-H, Fixed, 95% CI
ACTIV-4B, 2021	0	135	0	136		Not estimable
Ananworanich et al, 2021	0	222	0	222		Not estimable
MICHELLE，2022	0	159	4	159	18.4%	0.11 [0.01, 2.05]
CARE—COALITION VIII，2023	6	327	9	330	36.6%	0.67 [0.24, 1.87]
ACTIV-4C, 2023	8	610	9	607	36.9%	0.88 [0.34, 2.28]
PREVENT-HD, 2023	2	641	2	643	8.2%	1.00 [0.14, 7.10]
**Total (95% CI)**		**2094**		**2097**	**100.0%**	**0.67 [0.36, 1.25]**
Total events	16		24			

Heterogeneity: Chi² = 1.95, df = 3 (P = 0.58); I² = 0%
Test for overall effect: Z = 1.25 (P = 0.21)

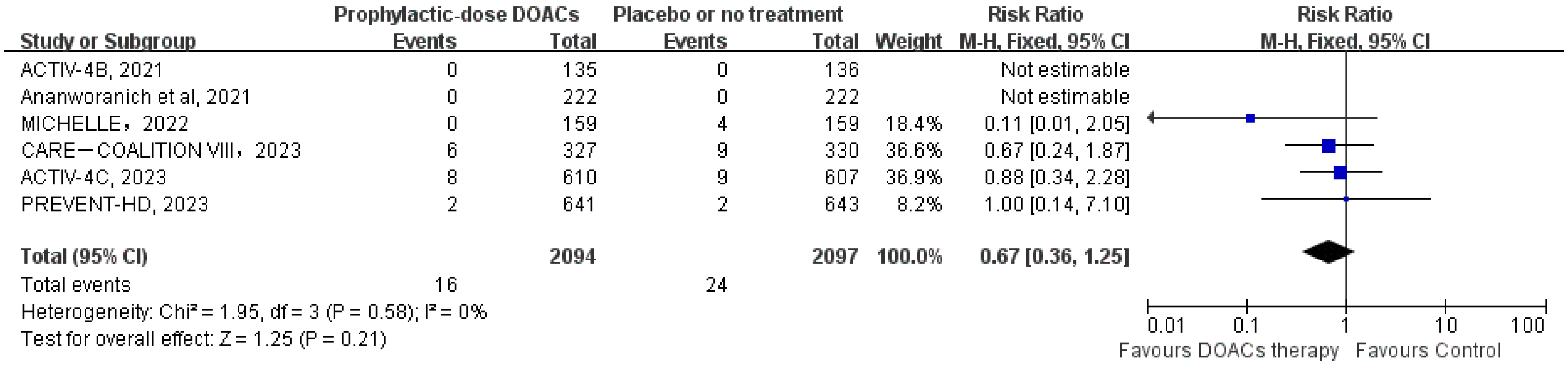

Figure S2. Forest plot illustrating the association of prophylactic-dose DOACs with all-cause mortality in non-hospitalized patients with COVID-19. DOACs, direct oral anticoagulants.

Study or Subgroup	Prophylactic-dose DOACs Events	Prophylactic-dose DOACs Total	Placebo or no treatment Events	Placebo or no treatment Total	Weight	Risk Ratio M-H, Fixed, 95% CI
ACTIV-4B, 2021	0	135	0	136		Not estimable
ACTIV-4C, 2023	5	610	5	607	19.3%	1.00 [0.29, 3.42]
CARE—COALITION VIII，2023	0	327	4	330	17.2%	0.11 [0.01, 2.07]
MICHELLE，2022	5	159	13	159	50.0%	0.38 [0.14, 1.05]
PREVENT-HD, 2023	0	641	3	641	13.5%	0.14 [0.01, 2.76]
**Total (95% CI)**		**1872**		**1873**	**100.0%**	**0.42 [0.21, 0.85]**
Total events	10		25			

Heterogeneity: Chi² = 3.19, df = 3 (P = 0.36); I² = 6%
Test for overall effect: Z = 2.42 (P = 0.02)

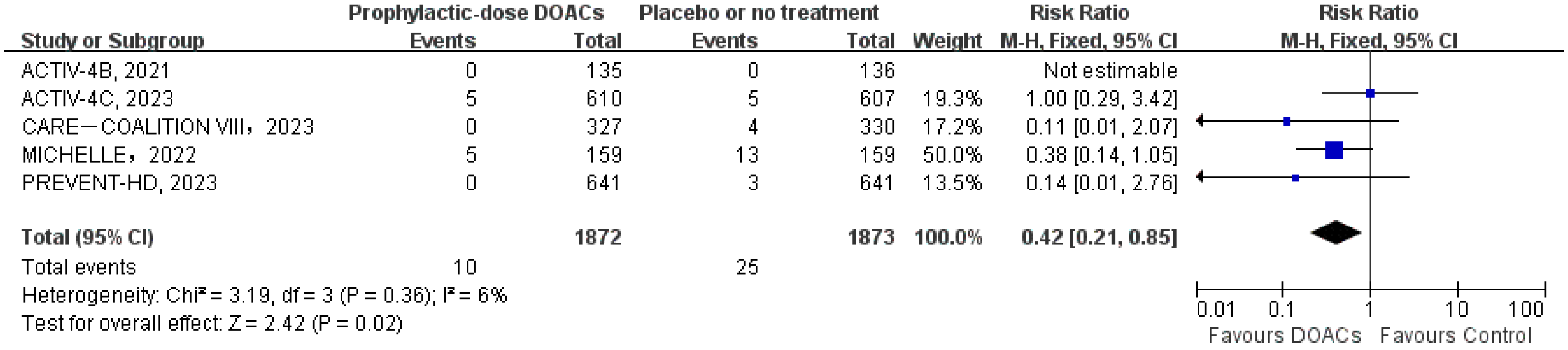

Figure S3. Forest plot illustrating the association of prophylactic-dose DOACs with VTE events in non-hospitalized patients with COVID-19. DOACs, direct oral anticoagulants; VTE, Venous thromboembolism.

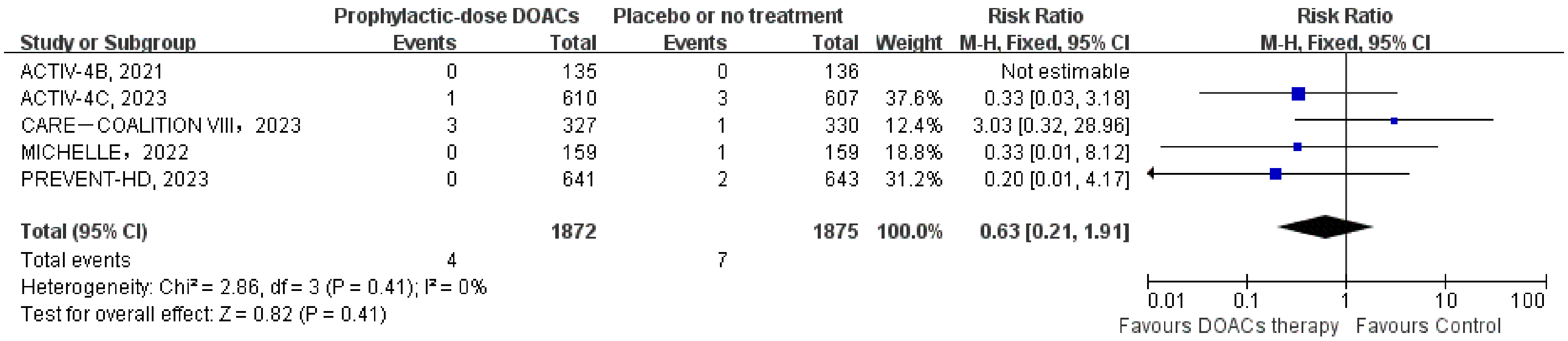

Figure S4. Forest plot illustrating the association of prophylactic-dose DOACs with ATE events in non-hospitalized patients with COVID-19. DOACs, direct oral anticoagulants; ATE, arterial thromboembolism.

	Prophylactic-dose DOACs		Placebo or no treatment			Risk Ratio
Study or Subgroup	Events	Total	Events	Total	Weight	M-H, Fixed, 95% CI
ACTIV-4B, 2021	1	135	0	136	0.9%	3.02 [0.12, 73.53]
Ananworanich et al, 2021	3	222	7	222	12.4%	0.43 [0.11, 1.64]
CARE—COALITION VIII，2023	36	327	32	330	56.6%	1.14 [0.72, 1.78]
PREVENT-HD, 2023	21	641	17	643	30.1%	1.24 [0.66, 2.33]
**Total (95% CI)**		**1325**		**1331**	**100.0%**	**1.10 [0.77, 1.55]**
Total events	61		56			
Heterogeneity: Chi² = 2.44, df = 3 (P = 0.49); I² = 0%						
Test for overall effect: Z = 0.51 (P = 0.61)						

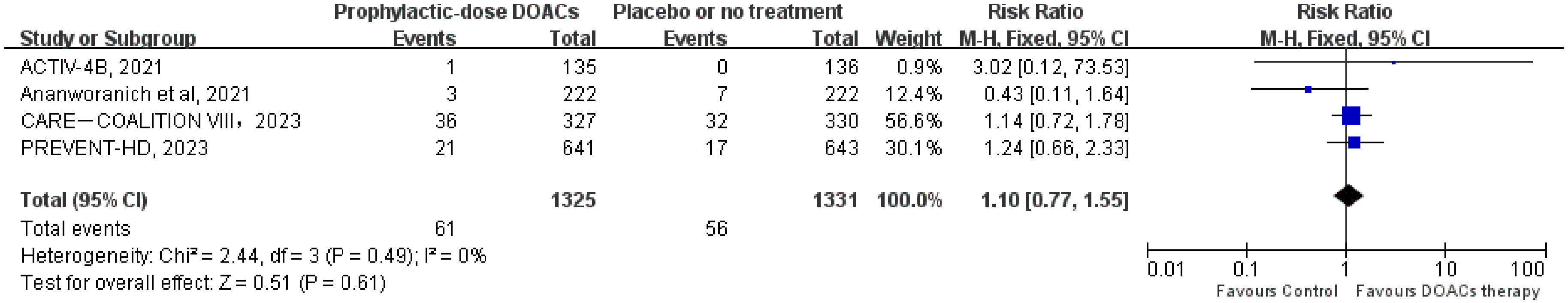

Figure S5. Forest plot illustrating the association of prophylactic-dose DOACs with hospitalizations in non-hospitalized patients with COVID-19. DOACs, direct oral anticoagulants;

Study or Subgroup	Prophylactic-dose DOACs Events	Total	Placebo or no treatment Events	Total	Weight	Risk Ratio M-H, Fixed, 95% CI
ACTIV-4B, 2021	1	135	0	136	4.3%	3.02 [0.12, 73.53]
ACTIV-4C, 2023	3	610	6	607	52.3%	0.50 [0.13, 1.98]
Ananworanich et al, 2021	5	222	2	222	17.4%	2.50 [0.49, 12.75]
MICHELLE，2022	2	159	2	159	17.4%	1.00 [0.14, 7.01]
PREVENT-HD, 2023	9	641	1	643	8.7%	9.03 [1.15, 71.05]
**Total (95% CI)**		**1767**		**1767**	**100.0%**	**1.78 [0.87, 3.66]**
Total events	20		11			
Heterogeneity: Chi² = 6.26, df = 4 (P = 0.18); I² = 36%						
Test for overall effect: Z = 1.58 (P = 0.12)						

Risk Ratio
M-H, Fixed, 95% CI

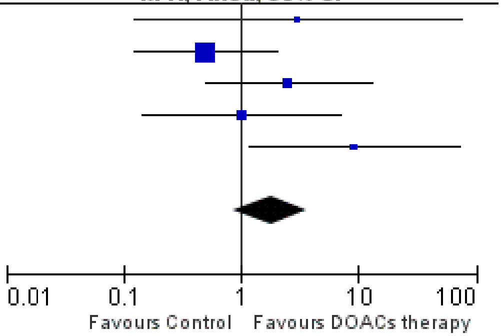

Figure S6. Forest plot illustrating the association of prophylactic-dose DOACs with clinically relevant nonmajor bleeding events in non-hospitalized patients with COVID-19. DOACs, direct oral anticoagulants
